# Female Genital Mutilation/Cutting: The Secret World of Women as Seen by Men

**DOI:** 10.1155/2013/643780

**Published:** 2013-07-10

**Authors:** Adriana Kaplan, Babucarr Cham, Lamin A. Njie, Ana Seixas, Sandra Blanco, Mireia Utzet

**Affiliations:** ^1^Department of Social and Cultural Anthropology, Universitat Autònoma de Barcelona, Belatterra 08193, Spain; ^2^Interdisciplinary Group for the Prevention and Study of Harmful Traditional Practices (IGPS/HTP), Department of Social and Cultural Anthropology, Universitat Autònoma de Barcelona, Belatterra 08193, Spain; ^3^NGO Wassu Gambia Kafo, Fajara F Section, Banjul, Gambia; ^4^School for Enrolled Community Health Nurses and Midwives, Mansakonko, Gambia; ^5^Group for Research in Africa and Latin America, Biostatistics Department, School of Medicine, Universitat Autònoma de Barcelona, Belatterra 08193, Spain

## Abstract

Efforts aimed at the abandonment of Female Genital Mutilation/Cutting (FGM/C) in the communities where it is deeply rooted have extensively considered and addressed women's perceptions on the issue, leaving those of men barely acknowledged. Although the practice is generally confined to the secret world of women, it does not mean that men cannot be influential. Indeed, men can play an important role in prevention. In order to address this gap, and having as background an extensive ethnographic field work, a transversal descriptive study was designed to explore Gambian men's knowledge and attitudes towards FGM/C, as well as related practices in their family/household. Results show ethnic identity, more than religion, as the decisive shaping factor on how men conceive and value FGM/C. The greater support towards the practice is found among traditionally practicing groups. A substantial proportion of men intend to have it performed on their daughters, although reporting a low involvement in the decision making process, with very few taking alone the final decision. Only a minority is aware of FGM/C health consequences, but those who understand its negative impact on the health and well-being of girls and women are quite willing to play a role in its prevention.

## 1. Introduction

Female Genital Mutilation/Cutting (FGM/C) is defined by the World Health Organization (WHO) [1] as all procedures involving partial or total removal of the external female genitalia, or injury to the female genital organs, for nontherapeutic reasons. The WHO classifies the practice into four types: type I (clitoridectomy), type II (excision), and type III (infibulation) are ordered according to a growing level of severity, while type IV comprises all other harmful procedures performed on the female genitalia for nonmedical purposes (e.g., pricking, piercing, incising, scraping, and cauterization).

According to the WHO latest data, 140 million women and girls in the whole world are thought to have been subjected to the practice, and 3 million girls are at risk of having it performed every year. FGM/C constitutes an extreme form of discrimination and violation of the human rights of girls and women, with health consequences now acknowledged and documented. In the short term, the practice can result in shock, haemorrhage, infections, and psychological consequences, while in the long term it can lead to chronic pain, infections, keloids, fibrosis, primary infertility, increase in delivery complications, and psychological sequela/trauma [2–7].

FGM/C has been practiced for centuries, having acquired a deep cultural meaning. Under a shared vision of the world where life is understood in cycles, FGM/C had been linked with the moment in which a girl becomes a woman in many societies. During the rite of passage to adulthood, within a ceremony secretly kept from outsiders, especially men, initiates were taught about the cultural and social wealth of their community, as well as their roles and responsibilities as women, mothers and wives, establishing gender power relationships [8]. The physical cutting would be the proof that a girl was granted with all necessary teachings that make her worthy to belong to her community. FGM/C had become a synonym of cleanliness, femininity, beauty, and purity, a way to protect virginity, guarantee “family's honour,” and ensure marriageability [9, 10].

In The Gambia, the overall prevalence is estimated at 76.3% [11], meaning that it affects approximately 3 out of 4 women. However, this global figure obviates important discrepancies within regions and ethnic groups, as shown in Tables 1 and 2. 

Its impact for health has been assessed in two clinical studies conducted in-country by the first author of the present paper, which revealed that 1 out of 3 girls and women presented injuries as a consequence of the practice [12] and the risk of complications during delivery and for the newborn increased 4.5 times for women with FGM/C [13]. Whilst these girls and women will need specific medical care for decades to come, prevention is an urgent step. However, strategies need to be carefully designed in order to respect the deep cultural value of the practice within the communities where it is performed. 

Although traditionally the practice was part of the rite of passage to womanhood among certain ethnic groups, as extensively described in an ethnographic research conducted by the first author of this paper [8], over the past generation several changes have been occurring. In a recent study, Shell-Duncan et al. [14] found that the physical cutting is increasingly becoming divorced from the traditional ritual. FGM/C is not a condition to ensure marriageability, but mainly a way to facilitate entry into a social network and have access to social support and resources, with peer pressure playing a major role in its perpetuation.

In order to gather evidence to inform prevention strategies, many studies have focused in women's perception regarding the practice, but much is still unknown about the role played by men on its perpetuation. However, their perception of the “secret world of women” might bring important elements to understand the context in which the practice occurs, as well as enlighten effective ways to involve them in prevention. What lies under their support towards the practice? Do they establish a parallelism with male circumcision, the cutting-off of the penis' foreskin prepuce? Indeed, in all the societies where FGM/C is found, male circumcision is also performed [15], sometimes linked to the rite of passage to adulthood as a keystone component of the socialization process. It has a similar hygienic and aesthetic meaning and an analogous power to preserve ethnic and gender identities [2, 3, 8, 16]. A deep situation analysis on FGM/C conducted in The Gambia in 1999 [17] revealed that some respondents established a parallelism between the two practices. Since Islam endorses male circumcision as an acceptable practice and makes no distinction between genders, some would argue that female circumcision is also prescribed. 

Acknowledging this gap, a new line of research is now emerging, interested in exploring how men position themselves on the matter, with the objective of assessing their potential inclusion in preventive actions and programmes. The results obtained so far have showed different—and sometimes contradictory—levels of involvement and support towards FGM/C that seem to be influenced by sociodemographic variables, such as ethnicity and religion [18–20]. Others have highlighted that both men and women blame each other for the continuation of the practice and position themselves as victims [21]. In a recent study conducted in The Gambia with health care professionals [22], it was discovered that FGM/C found higher support among men. While women would give more strength to the deep cultural roots of the tradition, men seemed to privilege a moral perspective, prioritizing the fact that the practice is mandatory by religion and attenuates women's sexual feelings, contributing to family honour.

This study intends to contribute towards this field of research, by exploring the knowledge and attitudes of Gambian men towards FGM/C, as well as practices in their family and household. It expects to help to increase the understanding of the social environment embedding the practice, in order to inform prevention strategies that might successfully accelerate its abandonment.

## 2. Materials and Methods

### 2.1. Design of the Study

A transversal descriptive study was designed with the main objective of assessing the knowledge and attitudes of Gambian men on FGM/C, as well as related practices in their family/household, exploring eventual associations with sociodemographic characteristics. 

A secondary objective was to empower and promote knowledge's ownership of the native population, through a strategy designed to build capacities on FGM/C and social research skills. For this reason, the study was integrated in the Practicum of Community Medicine of the School for Enrolled Community Health Nurses and Midwives (ECHN/M) at Mansakonko, Lower River Region. Students were given the responsibility for data collection, under the supervision of their tutors and trainers from Wassu Gambia Kafo (WGK), the non-governmental organization that supported the study.

To ensure the accuracy of this process, students received specific training on social research skills, by a team consisting of a medical anthropologist and ECHN/M tutors. Furthermore, prior to their involvement on this study, students had already been trained on FGM/C identification, management and prevention, as their school is one of the health schools that integrated FGM/C in its Academic Curriculum—an initiative of WGK. 

The survey was implemented through questionnaires administered face to face. Taking into consideration the sensitivity of the topic, it was considered that the best strategy to avoid resistance was to administer the questionnaires in the communities where these students were doing their practicum, and in their home villages. In this way, it was ensured that (1) they were known and respected; (2) shared the same cultural background of the interviewees; and (3) were able to speak their local language, what contributed to create an environment of trust conducive to conduct the interviews. The selection of the communities where the practicum was conducted was a responsibility of ECHN/M tutors. 

As a consequence of this strategy, the survey was implemented in three regions of the country: Lower River Region, North Bank Region, and West Coast Region. According to Census 2003, the population in the first two regions is predominantly rural (80% approximately), while in West Coast Region is mainly urban (60%) [23]. As stated in Table 1, FGM/C prevalence rates in these regions are 90.6%, 49.2%, and 84.5%, respectively [11].

### 2.2. Research Population

The overall sample is composed of 993 men. The study intended to capture men with heterogeneous profiles in terms of occupation, age, ethnicity, religion, and marital status, both from rural and urban areas. Due to the fact that this study was integrated on a strategy to build students and tutors capacities on social research, it was considered that a quota sampling method was the most feasible method to apply. Each student was requested to administrate the questionnaire to 30 men. 

### 2.3. KAP Questionnaire

The data collection tool was a questionnaire with nineteen close-ended questions, designed to gather information on men's knowledge and attitudes with regard to FGM/C, related practices in their families/households, and sociodemographic data. 

The questionnaire was developed by a researcher and medical anthropologist, having as background former ethnographic studies conducted in the country since 1989 [8].

Although the questionnaire was drawn up in English, the official language of The Gambia, students were carefully instructed to know how to administer it in local languages whenever needed, in order to ensure an accurate understanding of the questions and of what was meant by “FGM/C.” In The Gambia, the practice is generally conceived as the equivalent to types I and II as established by WHO, which are the most prevalent in the country (66.2% and 26.3%, resp., [12]). Each ethnic group has specific words to distinguish the “cutting” and the “sealing” formed during the healing process after cutting and repositioning the labia. 

### 2.4. Variables

The five socio-demographic variables comprised occupation (agriculture, livestock, and fishery sector; services sector; health professionals; education professionals; students), age, ethnic group (Mandinka, Wolof, Fula, Djola, Serahule, and Serer), religion (Muslim, Christian), and marital status (married, single). The variables analyzed, chosen from the questionnaire, are presented below. Among them, Q1, Q5, Q8, Q13, and Q15 were selected as active variables for the Cluster Analysis.

- *Q1*. Is FGM/C practiced in your family/household? (Yes/No)
- *Q3*. At what age is FGM/C done on girls in your family/household? (0–3/Above 4)
- *Q5*. Do you take part in the decision making process on FGM/C? (Yes/No)
- *Q6*. Who takes the final decision to practice FGM/C on your daughter? (Men/Women/Both men and women/Other relatives, community members)
- *Q8*. Do you know of any health consequences related to FGM/C? (Yes/No)
- *Q9*. Is FGM/C a mandatory practice by religion? (Yes/No)
- *Q10*. Is FGM/C equivalent to male circumcision? (Yes/No)
- *Q13*. Do you think men have a role to play in preventing FGM/C? (Yes/No)
- *Q15*. If you have a daughter in the future, do you intend to circumcise her? (Yes/No)
- *Q16*. Do you think that the practice of FGM/C should continue? (Yes/No).

### 2.5. Ethical Aspects

The study was submitted and approved by The Gambia Government/Medical Research Council Laboratories Joint Ethics Committee (Ref: R08002). The purpose of the research was carefully explained and clarified by the students to the interviewees. The administration of the questionnaires only took place after respondents' signature or thumb print on an informed consent that was kept under the custody of WGK. The identity of the participants was maintained through rigorous confidentiality. 

### 2.6. Statistical Analysis

A descriptive analysis was carried out of the main variables, and prevalence proportions (%) and 95% confidence intervals (95%  CI) were calculated for the overall sample and, in order to detect differences, for each of the socio-demographic variables (occupation, age, ethnic group, religion, and marital status). Prevalence proportions were compared with Chi-squared test or Fisher's exact test when appropriate. Unspecified data (“other religion” and “other ethnic group”) were not taken into account in the analysis.

Statistically significant differences were considered at *P* < 0.05. 

A multiple correspondence analysis (MCA) and a cluster analysis were conducted to detect underlying groups of individuals according to their knowledge and attitudes regarding FGM/C, as well as related practices in their families/households, as defined by the active variables. The five socio-demographic variables were included as supplementary information, allowing the identification of opposite profiles of men towards the practice.

The information was computerized via EpiData. Descriptive univariate and bivariate analyses were conducted through SPSS Version 19, while MCA and cluster analysis through SPAD version 5.6.

### 2.7. Methodological Issues

The main methodological issue regarding this study has to do with the sensitivity of the topic itself, as it is common to find resistance to talk openly about FGM/C, especially to an outsider. This was addressed by giving Gambian students the responsibility for interviewing people in communities where they were known and respected. Another methodological issue is related to the fact that Serahule's sample size was quite small (only 12 individuals).

## 3. Results

The socio-demographic characteristics of the respondents are shown in Table 5. The sample was composed predominantly of young men, their mean age being 36.5 years old, with Muslim affiliation (96.2%). The majority were married (74.4%) and worked in agriculture, livestock, and fishery (51.3%) or in services sector (20.6%). However, the sample also included education and health care professionals (7.8% and 7.0%, resp.) and a few students (7.6%). With regard to ethnicity, 41.2% were Mandinka, 19.9% Wolof, 17.6% Fula, 9.7% Djola, and 1.2% Serahule. 

The prevalence proportions and 95% CI of knowledge, attitudes, and practices, according to socio-demographic variables, are presented in Table 3. FGM/C appears in this study as a widespread practice, with a prevalence rate (70.0%) not far from the most recent official data (76.3%). A total of 61.8% men embrace its continuation and 60.7% intend to have it performed on their daughters in the future. Although FGM/C is mainly performed by families affiliated with Islam (72.5% versus 27.3% Christians, *P* < 0.05), prevalence proportions disagree amongst Muslims with different ethnic backgrounds. With statistically significant differences, traditionally practicing groups (Mandinka, Djola, Fula, and Serahule) are the ones reporting the highest prevalence rates in their families/households, expressing the highest support towards the continuation of the practice and the strongest willingness to have it performed on their daughters.

Also with statistically significant differences, almost 60% of Mandinka consider FGM/C as equivalent to men's circumcision, a parallelism that is established by 47.3% Djola, 43.8% Fula and 33.3% Serahule. Whilst 75% Serahule and 72.8% Mandinka believe that the practice is mandatory by Islam, only 56.0% Fula, and 36.4% Djola do so. Serer and Wolof, which are also Muslims but traditionally nonpracticing groups, do not establish a connection between the practice and Islam neither acknowledge a parallelism between FGM/C and male circumcision—indeed, around 95% of Wolof and 90% of Serer deny it (*P* < 0.05). Interesting but not statistically significant, men over 60 years old establish the relation between FGM/C and Islam and its equivalence with male circumcision in a higher percentage than other age groups.

In the overall sample, almost 72.0% of men do not know that FGM/C has a negative impact on the health and well-being of girls and women. The highest awareness is found among Wolof men (47.9%, *P* < 0.05), health and education professionals (48.0% and 46.3%, *P* < 0.05). Although not being a statistically significant trend, it is found that awareness of FGM/C health consequences decreases with age, with the lower levels being found among men over 60 years old (15.4%). Also interesting but not statistically significant is to find that the group of men between 31 and 45, who have the highest awareness of FGM/C health consequences, are also the less supportive of the practice, with a lower intention to have it performed on their daughters and the highest willingness of seeing men intervening in its prevention. The negative impact that the practice has on the health and welfare of girls and women is, indeed, the major reason given by 72.9% of those who, on the overall sample, are against its perpetuation.

This study also reveals that over 39.8% of girls are subjected to FGM/C before completing their fourth anniversary. This is mainly reported by men between 31 and 45, whilst men above 60 report the practice to occur when the girl child has already completed 4 years old (67.4%, *P* < 0.05). 

A minority of men take part in this decision-making process, especially if they are not married (married 39.3%, single 21.1%, *P* < 0.05). Only 8.0% take the final decision towards subjecting their daughters to the practice, and 6.2% join the wives in this decision (Table 4). FGM/C appears mainly as a women's choice (75.8%) or a decision of other relatives and community members (10.0%). Since there is no statistically significant association with the socio-demographic variables, this information is not shown in Table 4.

**Figure 1:**
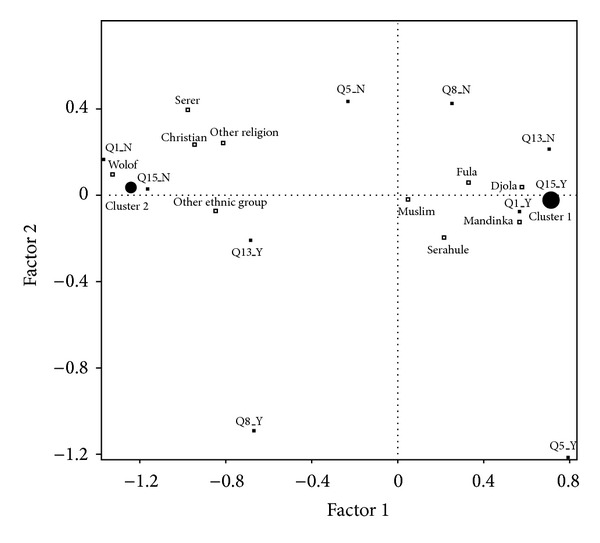
Multiple correspondence analysis.

*Cluster Analysis*. The cluster analysis revealed statistically significant differences for ethnicity and religious affiliation, allowing the identification of two profiles of respondents which are identified in Clusters 1 and 2 (Tables 5 and 6, and Figure 1). 

Cluster 1 is composed of those men who declare, on a rate statistically significant and higher than the overall sample, that FGM/C is practiced in their families/households (99.7% versus 67.4%); that they are involved in the decision making process (37.0% versus 25.6%); intend to have it performed on their own daughters (92.5% versus 60.9%); are not aware of the practice having health consequences (82.9% versus 71.7%); and do not think that men have a role to play in its prevention (68.8% versus 48.4%). This cluster comprises almost two-thirds of the overall sample (65.1%) and is overrepresented by men from Mandinka, Fula, Serahule, and Djola ethnic origins, with Muslim affiliation.

Cluster 2 comprises the remaining one-third of the total sample and is composed of those men whose knowledge, attitudes, and practices are opposite to the ones expressed by men in Cluster 1. This group gathers those who report, on a rate statistically significant and lower than the overall sample, that FGM/C is not practiced in their family/households (80.7% versus 30.0%); that they are not involved in the decision making process (87.2% versus 74.4%); do not intent to have it performed on their daughters (96.0% versus 39.1%); are aware that the practice has health consequences (47.0% versus 28.3%); and believe that men have a role to play in its prevention (86.1% versus 51.6%). In this group, Wolof and Serer ethnic origins are overrepresented, together with the Christian religion (7.5% versus 3.5%). 

## 4. Discussion

Seen through men's eyes, the secret world of women remains embedded in cloudy concepts shaped by culture in ethnic tradition, also influenced by religion. All ethnicities included in this study follow Islam, but each one of them establishes a different relation between FGM/C and religion. While those from traditionally practicing groups tend to consider the practice as a religious injunction or as “Sunna,” finding justification for its continuation, almost all those from traditionally nonpracticing groups deny that the practice is an obligation in Islam. FGM/C is, in fact, a pre-Islamic practice. 

Even within traditionally practicing groups, perceptions diverge substantially. Mandinka found FGM/C on its mandatory character by Islam and are eager to consider it as equivalent to male circumcision. Serahule share the same religious conviction but do not establish the equivalence with the male practice, in opposition to Djola, for whom religion does not seem to be significant but the parallelism with male circumcision is more evident. Although sharing the same nationality and religion, ethnic identities are built up on different cultural values and social norms, which are the decisive shaping factors of men's concept of the practice. Ethnicity's power to influence the knowledge, attitudes, and practices with regard to FGM/C had already been shown in a previous study conducted with Gambian health care professionals, by the same authors of this paper [22].

Amongst older men, FGM/C is seen as a mandatory practice by religion, equivalent to male circumcision, with no health consequences. But a window of opportunity for change is found among younger generations. Men between 31 and 45 are the less supportive towards the practice, have the lowest intention to have it performed on their daughters and the highest willingness to play a role in its prevention, and are also the group more aware of FGM/C health consequences. Can this increased knowledge and less supportive attitudes be linked, and on this foundation built on a strategy for prevention? This and other findings from this study suggest that it can. Indeed, among the group of men who are against the continuation of the practice, health consequences are presented as the major reason to stop its continuation. Health and education professionals, who are the ones more aware of FGM/C health consequences, show more willingness to participate in prevention. 

The fact that the majority of men are not active in the decision making process concerning the practice does not mean that they do not have the power to influence it. The finding that 60.7% of men intend to have FGM/C performed on their daughters in the future, but only 34.8% actually participate in the decision-making process and a few 14.2% take the final decision, alone (8.0%) or with their wives (6.2%), suggests that decision-making is not a simple one-way process. Indeed, field work evidence reveals that women who decide that their daughters will not undergo the practice face, not only peer pressure, but also feelings of helplessness when not actively supported by their husbands, as well as other influential male leaders from their communities. In a patriarchal society, although men might not be actively participating in FGM/C decision making process, they are still decision-makers.

The finding that decisions concerning FGM/C can be made by multiple actors including women, men, relatives, and community members corroborates the results achieved by Shell-Duncan et al. in a study recently conducted in The Gambia and Senegal [14]. These authors explain that the multiplicity of decision makers and peer pressure among women makes individuals less able to act upon intentions to carry on with the practice or not. In the secret world of women, avoiding discrimination is a powerful motif to perpetuate FGM/C, and this social force must be acknowledged. However, men's power to influence it should also not be disregarded.

Over the past generation, FGM/C practices have changed in many ways in Gambian societies. The group ritual in the “bush” is giving place to individual ceremonies behind doors [14]. Field experience reveals that the traditional knife, used to perform FGM/C on a number of girls without being sterilized, is being replaced with individual razor blades, as a result of HIV/AIDS awareness campaigns. Similarly, traditional herbs and charms, used to manage bleeding, relief pain and accelerate the healing process, are being complemented with modern drugs. Nowadays some babies and girls are taken to health facilities when health complications cannot be managed at community level, in opposition to the secrecy that characterized the seclusion period in the past. Sometimes, FGM/C is even performed by health professionals themselves: medicalization is already a reality in the country [22]. Finally, the age at which the practice is performed is declining—our study reveals that over 40% of Gambian girls are subjected to FGM/C before celebrating their fourth anniversary. This reduction may be explained by the belief that wounds heal faster and pain is lower for babies than for grown-up girls. 

This paper suggests that new actors can be called on stage to play an important role in FGM/C prevention. May knowledge be shared and synergies be built up, in order to promote positive changes that lead to the abandonment of the practice.

## 5. Conclusions 

Although sharing the same religious beliefs, men from traditionally and nontraditionally practicing groups see the relation between the practice and Islam in different ways and have diverse perceptions of its parallelism with male circumcision. Differences are also significant within traditionally practicing groups, showing how ethnic identities are the decisive shaping factors on how men conceive and value FGM/C. 

The decision to subject or not a girl to the practice appears as the result of a complex process involving multiple actors. Although few men are active participants in this process, their intention to have FGM/C performed on their daughters is likely to influence it. The support towards the practice is highly dependent on ethnic identity, being much higher among men from traditionally practicing groups. However, awareness on FGM/C health complications is prone to positively influence men's willingness to play a role in its prevention. In this line of thought, a strategy of acknowledging men's ethnic background and focusing on increasing their understanding of FGM/C negative impact on health might well be an effective way to influence and promote a positive change to the secret world of women.

## Figures and Tables

**Table 1:** FGM/C prevalence rates per region/LGA.

Region	Local government area	Prevalence
Great Banjul Area	Banjul	56.3%
	Kanifing	69.5%
West Coast Region	Brikama	84.5%
Lower River Region	Mansakonko	90.6%
North Bank Region	Kerewan	49.2%
Central River Region	Kuntaur	63.4%
	Janjanbureh	75.9%
Upper River Region	Basse	99.0%

Source: MICS2010, UNICEF The Gambia—GBOs.

**Table 2:** FGM/C prevalence rates per ethnic group.

Ethnic group	Prevalence
Mandinka/Jahanka	96.7%
Wolof	12.4%
Djola/Karoninka	87.0%
Fula/Tukulor/Lorobo	87.3%
Serer	43.0%
Serahule	97.8%

Source: MICS2010, UNICEF The Gambia—GBOs.

**Table:** (a)

	Q1. Is FGM/C practiced in your family/household?^a^			Q3. At what age is FGM/C done on girls in your family/household?^b,c^			Q5. Do you take part in the decision making process on FGM/C in your family?^a,c^		
	*n*	%	(95% CI)	*n*	%	(95% CI)	*n*	%	(95% CI)
Total	694	70.0		251	39.8		241	34.8	
Occupation									
Agriculture, livestock, and fishery sector	316	64.1	(59.8; 68.4)	109	37.8	(32.2; 43.5)	109	34.6	(29.2; 40.0)
Services sector	151	75.9	(69.7; 82.1)	54	41.2	(32.7; 49.7)	60	39.7	(31.6; 47.9)
Health professionals	55	82.1	(72.2; 92.0)	20	40.0	(26.3; 53.7)	21	38.9	(24.7; 52.8)
Education professionals	50	66.7	(55.3; 78.0)	19	38.8	(25.0; 52.6)	15	30.0	(16.3; 43.7)
Students	56	76.7	(66.3; 87.1)	17	34.7	(21.2; 48.1)	11	19.6	(8.3; 30.9)
	*P* = 0.001			*P* = 0.973			*P* = 0.099		

Age									
16–30 years	294	72.4	(67.9; 76.9)	94	34.8	(29.1; 40.5)	67	22.9	(17.9; 27.8)
31–45 years	255	65.7	(60.9; 70.6)	110	48.7	(42.1; 55.2)	117	46.1	(39.7; 52.4)
46–60 years	94	68.6	(60.5; 76.7)	32	36.4	(26.2; 46.5)	45	47.4	(36.8; 57.9)
+60 years	51	85.0	(75.1; 94.9)	15	32.6	(18.9; 46.3)	12	24.0	(11.2; 36.8)
	*P* = 0.011			*P* = 0.009			*P* < 0.0001		

Ethnic group									
Mandinka	394	97.8	(96.2; 99.3)	159	44.8	(39.6; 50.0)	144	36.6	(61.8; 41.5)
Wolof	10	5.1	(1.8; 8.5)	3	33.3	(0.6; 66.1)	3	33.3	(7.5; 70.1)
Fula	153	89.0	(84.0; 93.9)	48	34.0	(26.2; 41.9)	49	32.0	(24.3; 39.7)
Serahule	10	83.3	(51.6; 97.9)	4	40.0	(7.9; 72.1)	4	40.0	(12.2; 73.8)
Djola	95	100	(96.2; 100.0)	26	29.9	(20.2; 39.6)	31	32.6	(22.7; 42.6)
Serer	10	17.2	(6.7; 27.8)	4	44.4	(9.9; 78.9)	3	30.0	(6.7; 35.2)
	*P* < 0.0001			*P* = 0.093			*P* = 0.910		

Religion									
Muslim	651	72.5	(69.5; 75.5)	242	40.8	(36.8; 44.8)	231	35.6	(31.8; 39.4)
Christian	9	27.3	(10.6; 44.0)	2	22.2	—	1	9.0	—
	*P* < 0.0001			*P* = 0.324			*P* = 0.127		

Marital status									
Married	483	69.1	(65.6; 72.6)	188	42.9	(38.3; 47.6)	189	39.3	(34.8; 73.8)
Single	175	72.9	(67.1; 78.7)	51	32.5	(25.1; 38.2)	37	21.1	(14.8; 27.5)
	*P* = 0.265			*P* = 0.022			*P* < 0.0001		

^a^To allow a better understanding of the results, the table only presents the percentage of men who answered “yes” to the question.

^b^To allow a better understanding of the results, the table only presents the percentage of men who answered “0–3 years old” to the question.

^c^Only answered if replied “yes” in Q1.

**Table:** (b)

	Q8. Do you know of any health consequences related to FGM/C?^a^			Q9. Is FGM/C a mandatory practice by religion?^a^			Q10. Is FGM/C equivalent to male circumcision?^a^		
	*n*	%	(95% CI)	*n*	%	(95% CI)	*n*	%	(95% CI)
Total	280	28.3		443	46.2		388	39.8	
Occupation									
Agriculture, livestock, and fishery sector	122	24.7	(20.7; 28.6)	196	41.4	(36.8; 45.9)	175	36.2	(31.8; 40.5)
Services sector	48	24.2	(18.0; 30.5)	108	56.0	(48.7; 63.2)	92	46.9	(39.7; 54.2)
Health professionals	31	46.3	(33.6; 59.0)	30	47.6	(34.5; 60.7)	26	39.4	(26.8; 51.9)
Education professionals	36	48.0	(36.0; 60.0)	33	44.6	(32.6; 56.6)	28	38.4	(26.5; 50.2)
Students	19	26.0	(15.3; 36.8)	30	41.7	(29.6; 53.7)	27	50.0	(35.7; 64.3)
	*P* < 0.001			*P* = 0.011			*P* = 0.081		

Age									
16–30 years	117	28.7	(24.2; 33.3)	178	45.3	(40.2; 50.3)	163	40.6	(35.7; 45.6)
31–45 years	122	31.5	(26.8; 36.3)	170	45.1	(39.9; 50.2)	142	37.4	(32.4; 42.4)
46–60 years	33	24.1	(16.6; 31.6)	64	49.2	(40.3; 58.2)	54	40.0	(31.4; 48.6)
+60 years	8	15.4	(4.6; 26.2)	31	53.4	(39.7; 67.1)	29	49.2	(35.5; 62.8)
	*P* = 0.20			*P* = 0.568			*P* = 0.360		

Ethnic group									
Mandinka	97	24.0	(19.7; 28.3)	283	72.8	(68.2; 77.3)	234	59.4	(54.4; 64.4)
Wolof	93	47.9	(40.7; 55.2)	9	4.7	(1.5; 8.0)	10	5.2	(1.8; 8.5)
Fula	33	19.2	(13.0; 25.4)	93	56.0	(48.2; 63.9)	74	43.8	(36.0; 51.6)
Serahule	4	33.3	(9.9; 65.1)	9	75	(42.8; 94.5)	4	33.3	(9.9; 65.1)
Djola	16	16.8	(8.8; 24.9)	32	36.4	(25.7; 47.0)	44	47.3	(36.6; 58.0)
Serer	12	20.7	(9.4; 32.0)	6	10.3	(1.6; 19.0)	5	8.6	(2.9; 19.0)
	*P* < 0.0001			*P* < 0.0001			*P* < 0.0001		

Religion									
Muslim	253	28.2	(25.2; 31.2)	423	48.7	(45.3; 52.1)	364	41.1	(37.8; 44.4)
Christian	11	33.3	(15.7; 50.9)	3	9.1	(1.9; 24.3)	6	18.8	(7.2; 36.4)
	*P* = 0.518			*P* < 0.0001			*P* = 0.011		

Marital status									
Married	199	28.5	(25.1; 31.9)	312	46.6	(42.8; 50.5)	271	39.4	(35.7; 43.2)
Single	66	27.4	(21.5; 33.2)	108	45.6	(39.0; 52.1)	95	39.7	(33.3; 46.2)
	*P* = 0.378			*P* = 0.777			*P* = 0.934		

^a^To allow a better understanding of the results, the table only presents the percentage of men who answered “yes” to the question.

**Table:** (c)

	Q13. Do you think men have a role to play in preventing FGM/C?^a^			Q15. If you have a daughter in the future, do you intend to circumcise her?^a^			Q16. Do you think that the practice of FGM/C should continue?^a^		
	*n*	%	(95% CI)	*n*	%	(95% CI)	*n*	%	(95% CI)
Total	510	51.6		583	60.9		605	61.8	
Occupation									
Agriculture, livestock, and fishery sector	252	51.2	(46.7; 55.7)	275	56.7	(52.2; 61.2)	284	58.4	(54.0; 62.9)
Services sector	95	47.7	(40.5; 54.9)	130	68.8	(61.9; 75.7)	137	69.2	(62.5; 75.9)
Health professionals	38	57.6	(44.9; 70.3)	38	63.3	(50.3; 76.4)	38	59.4	(46.6; 72.2)
Education professionals	48	65.3	(53.9; 76.8)	44	59.5	(47.6; 71.3)	45	60.8	(49.0; 72.6)
Students	30	41.1	(29.1; 53.1)	40	55.6	(43.4; 67.7)	43	58.9	(46.9; 70.9)
	*P* = 0.05			*P* = 0.019			*P* = 0.055		

Age									
16–30 years	192	47.3	(42.3; 52.3)	253	63.4	(58.6; 68.3)	262	65.0	(60.2; 69.8)
31–45 years	218	56.3	(51.3; 61.4)	214	57.1	(51.9; 62.2)	215	56.3	(51.2; 61.4)
46–60 years	70	51.1	(42.4; 59.8)	78	62.4	(53.5; 71.3)	89	66.4	(58.0; 74.8)
+60 years	30	50.8	(37.2; 64.5)	38	64.4	(51.3; 77.5)	39	66.1	(53.2; 79.0)
	*P* = 0.089			*P* = 0.286			*P* = 0.039		

Ethnic group									
Mandinka	151	37.5	(32.6; 42.3)	342	88.4	(85.0; 91.7)	345	87.1	(83.7; 90.6)
Wolof	163	83.6	(78.1; 89.0)	10	5.2	(1.8; 8.6)	18	9.2	(4.9; 13.6)
Fula	73	42.7	(35.0; 50.4)	121	72.0	(64.9; 79.1)	129	76.3	(69.6; 83.0)
Serahule	5	41.7	(15.2; 72.3)	8	66.7	(34.9; 90.1)	7	63.6	(30.8; 89.1)
Djola	34	35.8	(25.6; 46.0)	76	84.4	(76.4; 92.5)	78	83.0	(74.8; 91.1)
Serer	46	80.7	(69.6; 91.8)	9	16.4	(5.7; 27.1)	12	21.1	(9.6; 32.5)
	*P* < 0.0001			*P* < 0.0001			*P* < 0.0001		

Religion									
Muslim	445	49.7	(46.3; 53.0)	554	63.7	(60.4; 66.9)	574	64.6	(61.4; 67.8)
Christian	25	75.6	(59.6; 91.9)	5	15.6	(5.3; 32.8)	6	18.2	(7.0; 35.5)
	*P* = 0.003			*P* < 0.0001			*P* < 0.0001		

Marital status									
Married	360	51.7	(47.9; 55.4)	408	60.8	(57.0; 64.6)	419	61.0	(57.3; 64.7)
Single	124	51.7	(45.1; 58.2)	143	60.3	(53.9; 66.8)	152	63.3	(57.0; 69.6)
	*P* = 0.996			*P* = 0.899			*P* = 0.520		

^a^To allow a better understanding of the results, the table only presents the percentage of men who answered “yes” to the question.

**Table 4:** Knowledge, attitudes, and practices.

Q6. Who takes the final decision to practice FGM/C on your daughter?^a^		
	*n*	%
Men	53	8.0
Women	502	75.8
Both men and women	41	6.2
Other relatives/community members	66	10.0

Total	662	100.0

^a^Only answered if replied “yes” in Q1.

**Table 5:** Socio-demographic description of sample and clusters.

	Cluster 1 (*n* = 646; 65.1%)		Cluster 2 (*n* = 347; 34.9%)		Total sample (*n* = 993)	
	*N*	%	*N*	%	*N*	%
Age						
16–30 years	272	42.1	135	38.9	407	41.0
31–45 years	240	37.2	148	42.7	388	39.1
46–60 years	92	14.2	46	13.3	138	13.9
+60 years	42	6.5	18	5.2	60	6.0
Total	**646**	**100.0**	**347**	**100.0**	**993**	**100.0**
Occupation						
Agriculture, livestock, and fishery sector	295	47.0	200	59.3	495	51.3
Services sector	146	23.3	53	15.7	199	20.6
Health professionals	49	7.8	18	5.3	67	7.0
Education professionals	45	7.2	30	8.9	75	7.8
Students	48	7.7	25	7.4	73	7.6
Other	44	7.0	11	3.3	55	5.7
Total	**627**	**100.0**	**337**	**100.0**	**964**	**100.0**
Ethnic group						
Mandinka	382	59.8*	22	6.4	404	41.2
Wolof	9	1.4	186	54.4*	195	19.9
Fula	133	20.8*	40	11.7	173	17.6
Serahule	10	1.6	2	0.6	12	1.2
Djola	84	13.1*	11	3.2	95	9.7
Serer	9	1.4	49	14.3*	58	5.9
Other	12	1.9	32	9.4	44	4.5
Total	**639**	**100.0**	**342**	**100.0**	**981**	**100.0**
Religion						
Muslim	608	98.5*	292	91.5	900	96.2
Christian	9	1.5	24	7.5*	33	3.5
Other	0	0.0	3	0.9	3	0.3
Total	**617**	**100.0**	**319**	**100.0**	**936**	**100.0**
Marital status						
Married	450	73.8	250	75.5	700	74.4
Single	160	26.2	81	24.5	241	25.6
Total	**610**	**100.0**	**331**	**100.0**	**941**	**100.0**

*Occurrence is significantly overrepresented in the given cluster than in the whole sample (*P* < 0.001).

**Table 6:** Description of the clusters (active variables).

Variable label	Characteristic categories	Percent of category in cluster	Percent of category in sample
Cluster 1			
Q1. Is FGM/C practiced in your family/household?	Yes	99.7	70.0
Q5. Do you take part in the decision making process on FGM/C?	Yes	37.0	25.7
Q8. Do you know of any health consequences related to FGM/C?	No	82.9	71.4
Q13. Do you think men have a role to play in preventing FGM/C?	No	68.8	48.4
Q15. If you have a daughter in the future, do you intend to circumcise her?	Yes	92.5	60.7

Cluster 2			
Q1. Is FGM/C practiced in your family/household?	No	80.7	30.0
Q5. Do you take part in the decision making process on FGM/C?	No	87.2	74.3
Q8. Do you know of any health consequences related to FGM/C?	Yes	47.0	28.6
Q13. Do you think men have a role to play in preventing FGM/C?	Yes	86.1	51.6
Q15. If you have a daughter in the future, do you intend to circumcise her?	No	96.0	39.3

## References

[1] World Health Organization (2008). *Eliminating Female Genital Mutilation: An Interagency Statement. OHCHR, UNAIDS, UNDP, UNECA, UNESCO, UNFPA, UNHCR, UNICEF, UNIFEM*.

[2] Dare FO, Oboro VO, Fadiora SO, Orji EO, Sule-Odu AO, Olabode TO (2004). Female genital mutilation: an analysis of 522 cases in South-Western Nigeria. *Journal of Obstetrics and Gynaecology*.

[3] Behrendt A, Moritz S (2005). Posttraumatic stress disorder and memory problems after female genital mutilation. *American Journal of Psychiatry*.

[4] Alsibiani SA, Rouzi AA (2010). Sexual function in women with female genital mutilation. *Fertility and Sterility*.

[5] Morison L, Scherf C, Ekpo G (2001). The long-term reproductive health consequences of female genital cutting in rural Gambia: a community-based survey. *Tropical Medicine and International Health*.

[6] World Health Organization Study Group on Female Genital Mutilation and Obstetric Outcome (2006). Female genital mutilation and obstetric outcome: WHO collaborative prospective study in six African countries. *The Lancet*.

[7] Chibber R, El-Saleh E, El Harmi J (2011). Female circumcision: obstetrical and psychological sequelae continues unabated in the 21st century. *The Journal of Maternal-Fetal and Neonatal Medicine*.

[8] Kaplan A (1998). *From Senegambia to Catalonia: Acculturation and Social Integration Process*.

[9] Gage AJ, van Rossem R (2006). Attitudes toward the discontinuation of female genital cutting among men and women in Guinea. *International Journal of Gynecology and Obstetrics*.

[10] United Nations Children’s Fund (2010). *The Dynamics of Social Change Towards the Abandonment of Female Genital Mutilation/Cutting in Five African Countries*.

[11] Gambia Bureau of Statistics (2011). The Gambia multiple indicator cluster survey.

[12] Kaplan A, Hechavarría S, Martín M, Bonhoure I (2011). Health consequences of female genital mutilation/cutting in the Gambia, evidence into action. *Reproductive Health*.

[13] Kaplan A, Forbes M, Bonhoure I (2013). Female Genital Mutilation/Cutting (FGM/C) in The Gambia: long-term health consequences and complications during delivery and for the newborn. *International Journal of Women’s Health*.

[14] Shell-Duncan B, Wander K, Hernlund Y, Moreau A (2011). Dynamics of change in the practice of female genital cutting in Senegambia: testing predictions of social convention theory. *Social Science and Medicine*.

[15] Johnsdotter S (2012). Projected cultural histories of the cutting of female genitalia: a poor reflection as in a mirror. *History and Anthropology*.

[16] World Health Organization (WHO) and the Joint United Nations Programme on HIV/AIDS (UNAIDS) (2008). *Male Circumcision: Global Trends and Determinants of Prevalence, Safety and Acceptability*.

[17] Government of The Gambia (1999). *A Situational Analysis on Female Genital Mutilation in the Gambia*.

[18] Ogunlola IO, Orji EO, Owolabi AT (2003). Female genital mutilation and the unborn female child in southwest Nigeria. *Journal of Obstetrics and Gynaecology*.

[19] Abdalla M, Omer A, Elmusharaf K (2012). Female genital mutilation in Sudan: what do men think?. *Contraceptions*.

[20] Sakeah E, Beke A, Doctor HV, Hodgson AV (2006). Males’ preference for circumcised women in northern Ghana. *African Journal of Reproductive Health*.

[21] Berggren V, Musa Ahmed S, Hernlund Y, Johansson E, Habbani B, Edberg AK (2006). Being victims or beneficiaries? Perspectives on female genital cutting and reinfibulation in Sudan. *African Journal of Reproductive Health*.

[22] Kaplan A, Hechavarría S, Bernal M, Bonhoure I Knowledge, attitudes and practices regarding FGM/C among rural Gambian health care professionals: a transcultural study.

[23] Gambia Bureau of Statistics (GBoS) (2006). *The Gambia Atlas of 2003 Population and Housing Census*.

